# The research progress of perioperative non-pharmacological interventions on postoperative cognitive dysfunction: a narrative review

**DOI:** 10.3389/fneur.2024.1369821

**Published:** 2024-05-01

**Authors:** Li Zhao, Yiping Guo, Xuelei Zhou, Wei Mao, Hongyu Zhu, Linlin Chen, Xianchun Liu, Longyi Zhang, Ying Xie, Linji Li

**Affiliations:** ^1^Department of Anesthesiology, The Second Clinical Medical College, North Sichuan Medical College, Nanchong Central Hospital, Nanchong, China; ^2^School of Humanities and Management, Key Laboratory for Quality of Life and Psychological Assessment and Intervention, Guangdong Medical University, Dongguan, China; ^3^Nanchong Center for Disease Control and Prevention, Nanchong, China

**Keywords:** cognitive function training, non-pharmacological interventions, perioperative neurocognitive dysfunction, postoperative cognitive dysfunction, review

## Abstract

Postoperative cognitive dysfunction (POCD) is a common neurological complication in elderly patients after surgery and general anesthesia. The occurrence of POCD seriously affects the postoperative recovery of patients, and leads to prolonged hospital stay, reduced quality of life, increased medical costs, and even higher mortality. There is no definite and effective drug treatment for POCD. More evidence shows that perioperative non-pharmacological intervention can improve postoperative cognitive function and reduce the incidence of POCD. Therefore, our studies summarize the current non-pharmacological interventions of POCD from the aspects of cognitive training, physical activity, transcutaneous electrical acupoint stimulation, noninvasive brain stimulation, non-pharmacological sleep improvement, music therapy, environment, and multimodal combination Interventions, to provide more data for clinical application and research.

## 1 Introduction

With an aging population and advances in global healthcare, more elderly patients are undergoing surgical procedures, and neurocognitive impairment related to surgery and anesthesia has further attracted widespread attention (1). Postoperative Cognitive Dysfunction (POCD) is a common neurological complication after surgery and general anesthesia, which refers to the impairment and regression of cognitive function after surgery in patients who had no preoperative cognitive dysfunction. POCD often occurs in elderly patients and is characterized by impaired thinking, memory, orientation, and executive ability (2, 3). The incidence of POCD has been reported to be 7–35% after noncardiac surgery (4) and up to 30–80% 1 month after cardiac surgery (5, 6). The occurrence of POCD seriously affects the postoperative recovery of patients, and leads to prolonged hospital stay, reduced quality of life, increased medical costs, and even higher mortality (7).

However, the exact mechanism of POCD remains unclear, and current research has found that it may be associated with many risk factors, including age, cognitive status, concomitant chronic diseases, nutritional status, anesthesia, surgery, and pain (8–10). Current evidence suggests that drug interventions have an uncertain effect on cognitive impairment (11, 12), so non-pharmacological interventions for POCD may be an effective means of prevention and a focus for future research. Many randomized controlled trials (RCT) and meta-analyses have shown that non-pharmacological interventions have a positive effect on the improvement and prevention of POCD (13, 14). Therefore, our studies summarize the current non-pharmacological interventions of POCD from the aspects of cognitive training, physical activity, transcutaneous electrical acupoint stimulation, noninvasive brain stimulation, non-pharmacological sleep improvement, music therapy, environment, and multimodal combination Interventions, to provide more data for clinical application and research.

## 2 Methods

### 2.1 Search strategy

PubMed and Cochrane databases were searched for studies published through October 2023. The search terms used were as follows: (postoperative cognitive dysfunction OR POCD OR postoperative delirium OR POD OR Perioperative neurocognitive disorders OR cognitive function) AND (non-pharmacological OR cognitive training OR cognitive intervention OR physical activity OR exercise OR acupoint stimulation OR noninvasive brain stimulation OR repetitive transcranial magnetic stimulation OR transcranial direct current stimulation OR music OR sleep OR communication). Only studies written in English are considered. The results of the Pubmed search are provided in Supplementary file S1. We examined references to some of the studies to find other relevant papers. We did not present this study as a systematic literature review, but rather as a narrative review, given the great heterogeneity of different interventions and the variety of outcome measures reported in different studies.

### 2.2 Inclusion and exclusion criteria

The inclusion criteria were as follows: (1) The intervention group received one or more perioperative non-pharmacological interventions. (2) Outcome measures included incidence of POCD, cognitive function scores, or other evaluations of cognitive function. (3) This study does not restrict the study population. (4) There are no restrictions on the type of study, which can be randomized controlled trials, reviews, meta-analyses, etc.

We excluded studies from which data could not be extracted or used for analysis.

## 3 Results

### 3.1 Cognitive function training

Cognitive function training has been defined as a training program that provides structured practice on specific cognitive tasks to improve cognitive functioning by training patients in cognitive aspects such as memory, executive processing, language skills, attention, and perceptual awareness (15). Current studies believe that the mechanism of cognitive function training to prevent POCD may mainly be as follows: First, cognitive training may increase the density of cortical dopamine D1 receptor, and human cognitive function depends on adequate dopamine neurotransmission (16, 17). Secondly, some studies have found that cognitive training can improve cognitive reserve, and the incidence of POCD after surgery has been proven to be lower in patients with higher cognitive reserve (represented by a higher educational background). Therefore, cognitive training may improve POCD by improving cognitive reserve (18, 19). Third, previous studies have found that cognitive function, perception, and memory function will gradually decline during the aging process. Still, the brain retains lifelong plasticity and adaptive reorganization ability, so the use of appropriately designed training programs can improve some cognitive functions of the brain (20, 21). At present, there are various ways of cognitive function training. According to cognitive training tools, it can be divided into traditional cognitive training based on paper, pen, reading, reciting, etc., and cognitive training based on computer programs.

#### 3.1.1 Cognitive training based on traditional training methods

An RCT of 94 patients undergoing cardiac surgery by Butz et al. found that 36 min of paper and pen-based cognitive training per day for 3 weeks post-surgery significantly reduced the incidence of POCD and remained statistically different 15 months after the intervention (22). In an RCT of 141 elderly patients undergoing surgery for gastrointestinal tumors, Saleh et al. found that the incidence of POCD in elderly patients at 7 days postoperatively could be significantly reduced by preoperative spatial memory training (23). In addition, in an RCT of 72 elderly patients undergoing coronary artery bypass grafting, Li et al. found that preoperative training of patients in memory, orientation, language, and attention significantly reduced the incidence of POCD at two weeks postoperatively (24).

A large number of studies have confirmed that the traditional cognitive training approach can significantly improve postoperative cognitive function and reduce the incidence of POCD, especially in elderly patients. In addition, it has advantages in terms of convenience, economy, and practicality.

#### 3.1.2 Cognitive training based on computer programs

With the proliferation of computers, cognitive function training through the use of designed apps is gaining popularity. O’Gara et al.’s RCT of 40 patients undergoing cardiac surgery found that 30 min of daily training with a mobile phone app in the preoperative period and for 4 weeks postoperatively significantly reduced the incidence of POCD (25). Song et al.’s RCT of 42 lung transplant patients found that after eight consecutive weeks of 40-min-a-day computer-based cognitive training in three cognitive domains, including memory, attention, and processing speed, beginning at postoperative week 5. The cognitive training group scored significantly higher than the control group on both the numerical and verbal fluency tests when administered at 12 weeks post-intervention (26). However, an RCT of 52 elderly patients by Vlisides et al. found that after 20 min of home computer-based cognitive training per day for 1 week preoperatively, there were no statistically significant differences between the cognitive training group and controls when memory, attention, and processing speed were assessed at 3 days postoperatively (27). In addition, an RCT of 36 elderly patients undergoing coronary artery bypass grafting by Greaves et al. found that after 13 weeks of home computer-based cognitive training targeting 4 domains of attention, memory, reaction, and executive function, there were no statistical differences between the cognitive training group and the control group in any of the 4 cognitive domains at the time of discharge, at 4 months after discharge, and at the time of assessment 6 months later (28).

There is currently a large discrepancy in the outcome performance of POCD using cognitive training with computer programs, leading to the following possibilities for inconsistency in POCD outcomes. First, most of the study participants were elderly, who are usually not fluent with computers. Second, the cognitive training programs of Song et al. (26), Vlisides et al. (27), and Greaves et al. (28) were completed by the patients themselves at home rather than in the hospital using computers, which exacerbated the low compliance of the elderly patients. Therefore the effectiveness of the cognitive training was greatly diminished. The study by Vlisides et al. (27) mentioned that only 5 (out of 29) completed all the cognitive training, and the study by Humeidan et al. (29) mentioned that only 11 (out of 125) completed all the cognitive training, which confirmed the low adherence to computerized cognitive training in elderly patients. Thirdly, the sample sizes of the studies by Vlisides et al. (27) and Greaves et al. (28) were small, which reduced the strength of the evidence for the outcome. The use of computer programs has the advantage of portability compared to traditional cognitive training methods, but their utility and cognitive training effectiveness for older adults are controversial, so this needs to be validated in more RCTs.

### 3.2 Transcutaneous electrical acupoint stimulation

Transcutaneous electrical acupoint stimulation (TEAS) is an important part of traditional Chinese medicine in China (30). TEAS is an emerging acupuncture therapy guided by the meridian theory of Chinese medicine and combined with transcutaneous electrical nerve stimulation technology. TEAS uses noninvasive electrical stimulation technology to add perceptible pulsed stimulation to electrodes placed on the surface of acupoints. Unlike conventional acupuncture, TEAS does not puncture the skin and avoids the risk of infection to a large extent (31). TEAS can play a protective role in the brain by hindering microglia activation, reducing oxidative stress, inhibiting central and peripheral inflammatory responses, and lowering inflammatory factor levels (32).

Hua et al.’s RCT of 97 elderly patients undergoing thoracoscopic surgery found that after TEAS from 30 min before until the end of the procedure, the TEAS group had significantly higher scores on the mini-mental state examination (MMSE) scale than the control group on days 1, 3, 5, and 7 postoperatively (33). An RCT of 84 elderly patients undergoing total intravenous anesthesia by Wu et al. found that after TEAS during surgery, MMSE and Montreal cognitive assessment (MoCA) scores were significantly higher in the TEAS group than in the control group at 1, 3, and 7 days postoperatively (34). In addition, a meta-analysis including 13 RCTs with a total of 999 elderly patients found that the incidence of POCD in the TEAS group was significantly lower than that in the control group at 3 months postoperatively, and subgroup analyses showed that patients in the TEAS group had a lower incidence of POCD than those in the control group in both orthopedic and abdominal surgery (35). Zhang et al.’s meta-analysis including 29 RCTs with a total of 1994 patients similarly found that the incidence of POCD was significantly lower in the TEAS group than in the control group at 1, 3, and 7 days postoperatively (36).

It has been widely verified that TEAS can reduce the incidence of POCD, and it has the advantages of painlessness, parameter unification, simple operation, and safety, so it is worthy of extensive clinical promotion (37).

### 3.3 Noninvasive brain stimulation

Noninvasive brain stimulation may improve cognitive function by modulating the excitatory of cortical neurons, enhancing the sensory-motor network, the left fronto-parietal network, and the functions related to the level of consciousness (38, 39). There is evidence that noninvasive brain stimulation enhances cognitive abilities, including memory, attention, and perception (40, 41).

Repetitive transcranial magnetic stimulation (rTMS) and transcranial direct current stimulation (tDCS), as the two main noninvasive and safe brain stimulation techniques, are now widely used in the diagnosis and treatment of various neuropsychiatric disorders. A meta-analysis of 438 participants found that rTMS improved cognitive function for one month in patients with mild cognitive impairment and Alzheimer’s disease (42). Tao et al.’s RCT of 122 elderly patients undergoing orthopedic surgery found that 20 min of tDCS during tracheal extubation significantly reduced the incidence of postoperative delirium (POD) at 3 days postoperatively (43). Wu et al. selected POCD-susceptible mice for rTMS 10 days before surgery, and neurocognitive assessment on days 3 to 10 after surgery found that rTMS significantly improved postoperative cognitive decline and spatial memory impairment in mice (44).

There have been many studies demonstrating that noninvasive brain stimulation can improve cognitive function in patients with mild cognitive impairment and Alzheimer’s disease (45–47), but there is not enough data to support the prevention and treatment of POCD for the time being, which needs to be explored in more RCTs.

### 3.4 Physical activity

Physical activity is widely believed to have positive health benefits and is associated with improved cognitive function and brain health (48). Trubnikova et al.’s RCT of 97 patients undergoing coronary artery bypass grafting found that aerobic running for 5–7 days preoperatively significantly reduced the brain’s susceptibility to ischemia and lowered the incidence of POCD for 7–10 days postoperatively (49). In a retrospective study of 151 patients, Yanagisawa et al. found that low preoperative physical activity was an independent predictor of POD in patients with gastrointestinal tumors (50). Chapman et al. found that shorter periods of aerobic exercise can also aid in neurological recovery, improve cognitive function, and benefit brain health (51).

In general, although many hypotheses have been proposed regarding the improvement of cognitive function by physical activity (52), they have not been confirmed, and RCTs directly evaluating the effect of physical activity on POCD are relatively few. In addition, physical activity intensity and physical activity type have different effects on POCD improvement, and further research is needed to determine which exercise mode is more effective for POCD high-risk groups.

### 3.5 Non-pharmacological sleep improvement

Postoperative patients often suffer from sleep disturbances. Poor sleep quality is closely associated with the development of POCD, and improving sleep is an important part of POCD prevention strategies (53). Maintaining a quiet and dimly lit environment on the ward, reducing distractions from nighttime nursing activities, and using eye masks and earplugs are some common measures (54). A meta that included 1,455 patients found that the use of earplugs at night in ICU patients significantly reduced the incidence of delirium (55). Kamdar et al. found that using a combination of sleep-promoting measures for ICU patients significantly improved sleep disturbances and reduced the incidence of delirium. These measures included turning off the television, dimming the lights, reducing nursing activities, using earplugs, and eye masks, and playing soothing music (56). Menger et al. found that the use of earplugs in patients on the first night after cardiothoracic surgery significantly improved sleep quality as well as patient satisfaction, reduced pain intensity, facilitated postoperative recovery, and reduced hospitalization costs (57). However, some studies have found the opposite conclusion. Leong et al. found that the use of earplugs and eye masks did not help to improve sleep quality in 100 patients undergoing abdominal surgery 1–3 days after surgery (58).

For post-surgical patients, the effect of using non-pharmacological sleep interventions on improving POCD remains inconclusive. Studies directly examining the effects of nonpharmacologic sleep interventions on POCD are still scarce, so the effects of non-pharmacological sleep aids on POCD need to be further explored.

### 3.6 Music therapy

Music therapy is the use of music and rhythm to treat physical and psychological disorders (59). Music therapy has the function of regulating mood and stress and reducing the release of inflammatory factors (60). Khan et al.’s RCT of 200 coronary artery bypass grafts found that listening to music on headphones for 1 h per day for one week postoperatively significantly reduced the incidence of POD (60). An RCT of 115 surgical patients with type A aortic coarctation by Wu et al. found that Beck’s cognitive training combined with music therapy for two months postoperatively significantly reduced the incidence of POCD (61). A meta-analysis by Golubovic et al. found that music therapy can significantly reduce the incidence of POCD (62).

Music therapy has the feasibility of clinical application, with a simple and cost-effective process and objective and stable results, providing new evidence for perioperative non-pharmacological interventions to improve cognitive function. However, there are relatively few studies evaluating music therapy for POCD. In addition, the effect of the timing, duration, and choice of music intervention on POCD improvement remains unclear.

### 3.7 Environment

Enrich environment has profound effects on the central nervous system of adults. Several studies have found that an enrich environment alters animal behavior and improves cognitive function, especially learning and memory (63). In some animal model studies, it has been found that environmental states that provide animals with more sensory stimulation can increase their spatial memory capacity, and cognitive functioning (64, 65). However, studies investigating the Enrich environment and POCD in humans are scarce. For perioperative patients, an appropriate environment may improve postoperative cognitive function.

### 3.8 Multimodal combination of non-pharmacological interventions

To further improve the effect on postoperative cognitive function, many studies have used multiple intervention modalities. Some studies have explored the effects of cognitive training combined with physical activity on POCD. Duan et al.’s RCT of 86 elderly patients undergoing orthopedic surgery found that 30 min of reading-based cognitive training per day perioperative period combined with 40 min of physical activity per day postoperatively significantly reduced the incidence of POCD within 1 week postoperatively (66). Linying found that intellectual training combined with physical activity was effective in reducing the incidence of postoperative inflammatory factors and POCD in elderly patients undergoing fracture surgery (67).

Elderly people have special characteristics such as the coexistence of multiple diseases, and deterioration of body functions and cognitive functions, so the risk of POCD and its severity increase accordingly. The Hospital Elder Life Program (HELP) is a targeted, multidisciplinary, collaborative strategy that has been shown to be effective and cost-efficient in preventing delirium, cognitive decline, and other common complications in hospitalized older adults (68–70). HELP is a combination of multiple non-pharmacological interventions, including orientation training, cognitive training, early activity, sleep assistance, visual stimulation, auditory stimulation, dietary guidance, and early multidisciplinary consultation. A meta-analysis of 3,605 patients found that HELP-based interventions reduced the odds of delirium by 45% and significantly reduced the length of hospitalization (71). Wang et al. found that HELP was effective in reducing POD, improving postoperative cognitive function, and shortening the length of hospitalization in elderly patients (72). Liang et al. found in 140 elderly fracture patients that modified HELP significantly improved cognitive function at 1 and 12 months after surgery (73).

## 4 Limitations

This study had some limitations. First, the mechanisms by which many non-pharmacological interventions improve cognitive function remain unclear. Secondly, some studies have shown contrary results, and the effectiveness of interventions still needs to be validated in more large-sample randomized controlled trials. In addition, some interventions have less research data, which still needs to be validated in more studies. Another limitation is that only two databases were used for the literature search.

## 5 Conclusion

With the increasing demand for perioperative comfort treatment, more studies have begun to focus on postoperative complications (74). The pathogenesis of POCD, one of the most common postoperative complications, is not fully understood, and there are no specific drugs for POCD. Therefore, this study summarized the perioperative non-pharmacological interventions regarding POCD by reviewing the literature, including cognitive training, physical activity, transcutaneous electrical acupoint stimulation, noninvasive brain stimulation, non-pharmacological sleep improvement, music therapy, environment, and multimodal combination Interventions. All of these interventions have been shown to be effective in slowing disease progression and improving postoperative cognitive function (Table 1). In addition, non-pharmacological interventions have become a hot research topic in recent years because they are usually low-cost, easy to implement, and have few adverse effects. However, there is still controversy about the therapeutic effects of different studies on the same method. Influencing factors that lead to differences in efficacy may be different ages, literacy levels, intensity of the intervention, and outcome assessment indicators. In future studies, it is necessary to set more uniform study inclusion criteria to achieve higher comparability of efficacy. In addition, many studies have only evaluated the short-term cognitive function after surgery, and the long-term effects on cognitive function improvement have yet to be verified. Therefore, future studies can continue to explore the potential mechanisms of nonpharmacologic interventions for the prevention of POCD and investigate more forms of non-pharmacologic interventions, which will have a great deal of room for development and prospects.

**Table 1:** The non-pharmacological treatment for postoperative cognitive function.

Intervention	Author	Sample size (number)	Conclusion	Main positive outcomes	Limitations
Cognitive function training	①Saleh (23) ②O’Gara (25)	①141 ②40	①Reduce the incidence of POCD at 7 days postoperatively. ②Reduce the incidence of POCD at discharge.	Convenient, cost-effective, practical and remarkably effective.	Attention to older adults’ lack of proficiency in computer-based cognitive training.
Transcutaneous electrical acupoint stimulation	①Zhang (36)	①1994	①Reduce the incidence of POCD at 1, 3, and 7 days postoperatively.	Painless, easy to perform, safe and effective.	Mechanisms need to be further explored.
Noninvasive brain stimulation	①Xie (42) ②Tao (43)	①438 ②122	①rTMS improved cognitive function for one month in patients with mild cognitive impairment and Alzheimer’s disease. ②tDCS during tracheal extubation significantly reduced the incidence of POD at 3 days postoperatively.	It has the potential to improve postoperative cognition.	Clinical data related to POCD are scarce and require further validation.
Physical Activity	①Trubnikova (49) ②Yanagisawa (50)	①97 ②151	①Aerobic running for 5–7 days preoperatively significantly reduced the incidence of POCD for 7–10 days postoperatively. ②Low preoperative physical activity was an independent predictor of POD in patients with gastrointestinal tumors.	It has the potential to improve postoperative cognition.	The exact effects and mechanisms still need to be further explored.
Non-pharmacological sleep improvement	①Menger (57) ②Leong (58)	①63 ②100	①The use of earplugs in patients after cardiothoracic surgery significantly improved sleep quality, reduced pain intensity, and facilitated postoperative recovery. ②The use of earplugs and eye masks did not help to improve sleep quality undergoing abdominal surgery 1–3 days after surgery.	Several studies have demonstrated its effectiveness in improving cognitive function.	The conclusions vary from study to study and further exploration of the effects is still needed.
Music therapy	①Khan (60) ②Golubovic (62)	①200 ②499	①Listening to music on headphones for 1 hour per day for one week postoperatively significantly reduced the incidence of POD. ②music therapy can significantly reduce the incidence of POCD.	Feasibility, simplicity, low cost and significant results.	Little research data.
Environment	①Hua (65)	①Not applicable	①Environmental states that provide animals with more sensory stimulation can increase their spatial memory capacity, and cognitive functioning.	Animal experiments have demonstrated its effectiveness and mechanism.	Little research data.

rTMS, Repetitive transcranial magnetic stimulation. tDCS: transcranial direct current stimulation.

## Author contributions

LiZ: Writing – review & editing, Writing – original draft, Investigation, Conceptualization. YG: Writing – review & editing, Writing – original draft, Investigation, Conceptualization. XZ: Writing – original draft, Software, Methodology, Formal analysis, Data curation. WM: Writing – original draft, Software, Methodology, Formal analysis, Data curation. HZ: Writing – review & editing, Project administration, Formal analysis. LC: Writing – review & editing, Project administration, Formal analysis. XL: Writing – original draft, Methodology. LoZ: Writing – original draft, Methodology. YX: Writing – review & editing, Visualization, Validation, Supervision, Resources, Funding acquisition. LL: Writing – review & editing, Visualization, Validation, Supervision, Resources, Funding acquisition.

## References

[1] Safavynia SA Goldstein PA. The role of neuroinflammation in postoperative cognitive dysfunction: moving from hypothesis to treatment. Front Psych. (2018) 9:752. doi: 10.3389/fpsyt.2018.00752, PMID: 30705643 PMC6345198

[2] Evered L Silbert B Knopman DS Scott DA DeKosky ST Rasmussen LS . Recommendations for the nomenclature of cognitive change associated with anaesthesia and surgery-2018. Br J Anaesth. (2018) 121:1005–12. doi: 10.1016/j.bja.2017.11.087, PMID: 30336844 PMC7069032

[3] Terrando N Brzezinski M Degos V Eriksson LI Kramer JH Leung JM . Perioperative cognitive decline in the aging population. Mayo Clin Proc. (2011) 86:885–93. doi: 10.4065/mcp.2011.0332 21878601 PMC3257991

[4] Monk TG Weldon BC Garvan CW Dede DE van der Aa MT Heilman KM . Predictors of cognitive dysfunction after major noncardiac surgery. Anesthesiology. (2008) 108:18–30. doi: 10.1097/01.anes.0000296071.19434.1e, PMID: 18156878

[5] Kotekar N Shenkar A Nagaraj R. Postoperative cognitive dysfunction – current preventive strategies. Clin Interv Aging. (2018) 13:2267–73. doi: 10.2147/CIA.S133896, PMID: 30519008 PMC6233864

[6] Newman MF Mathew JP Grocott HP Mackensen GB Monk T Welsh-Bohmer KA . Central nervous system injury associated with cardiac surgery. Lancet (London, England). (2006) 368:694–703. doi: 10.1016/S0140-6736(06)69254-4 16920475

[7] Steinmetz J Christensen KB Lund T Lohse N Rasmussen LS. Long-term consequences of postoperative cognitive dysfunction. Anesthesiology. (2009) 110:548–55. doi: 10.1097/ALN.0b013e318195b569 19225398

[8] Kong H Xu L Wang D. Perioperative neurocognitive disorders: a narrative review focusing on diagnosis, prevention, and treatment. CNS Neurosci Ther. (2022) 28:1147–67. doi: 10.1111/cns.13873, PMID: 35652170 PMC9253756

[9] Evered L Atkins K Silbert B Scott DA. Acute peri-operative neurocognitive disorders: a narrative review. Anaesthesia. (2022) 77:34–42. doi: 10.1111/anae.15613, PMID: 35001385

[10] Xu X Hu Y Yan E Zhan G Liu C Yang C. Perioperative neurocognitive dysfunction: thinking from the gut? Aging. (2020) 12:15797–817. doi: 10.18632/aging.103738, PMID: 32805716 PMC7467368

[11] Li L Wang C Fang M Xu H Lu H Zhang H. Effects of dexamethasone on post-operative cognitive dysfunction and delirium in adults following general anaesthesia: a meta-analysis of randomised controlled trials. BMC Anesthesiol. (2019) 19:113. doi: 10.1186/s12871-019-0783-x, PMID: 31253079 PMC6599229

[12] Siddiqi N Stockdale R Britton AM Holmes J. Interventions for preventing delirium in hospitalised patients. Cochrane Database Syst Rev. (2007) 18:CD 5563. doi: 10.1002/14651858.CD005563.pub2 17443600

[13] Li Z Yiping G Xuelei Z Wei M Linji L. Preoperative cognitive training improves postoperative cognitive function: A meta-analysis and systematic review of randomized controlled trials. Front Neurol. (2024) 14:1293153. doi: 10.3389/fneur.2023.1293153, PMID: 38259656 PMC10800879

[14] Zhao L Zhu H Mao W Zhou X Xie Y Li L. Effects of perioperative cognitive function training on postoperative cognitive dysfunction and postoperative delirium: a systematic review and meta-analysis. Front Neurol. (2023) 14:1146164. doi: 10.3389/fneur.2023.1146164, PMID: 37416309 PMC10322196

[15] Guglietti B Hobbs D Collins-Praino LE. Optimizing cognitive training for the treatment of cognitive dysfunction in Parkinson's disease: current limitations and future directions. Front Aging Neurosci. (2021) 13:709484. doi: 10.3389/fnagi.2021.709484, PMID: 34720988 PMC8549481

[16] Bäckman L Lindenberger U Li S Nyberg L. Linking cognitive aging to alterations in dopamine neurotransmitter functioning: recent data and future avenues. Neurosci Biobehav Rev. (2010) 34:670–7. doi: 10.1016/j.neubiorev.2009.12.008, PMID: 20026186

[17] McNab F Varrone A Farde L Jucaite A Bystritsky P Forssberg H . Changes in cortical dopamine d1 receptor binding associated with cognitive training. Science (New York, N.Y.). (2009) 323:800–2. doi: 10.1126/science.1166102 19197069

[18] Feinkohl I Winterer G Spies CD Pischon T. Cognitive reserve and the risk of postoperative cognitive dysfunction. Dtsch Arztebl Int. (2017) 114:110–7. doi: 10.3238/arztebl.2017.0110 28302254 PMC5359463

[19] Petrelli A Kaesberg S Barbe MT Timmermann L Rosen JB Fink GR . Cognitive training in Parkinson's disease reduces cognitive decline in the long term. Eur J Neurol. (2015) 22:640–7. doi: 10.1111/ene.12621, PMID: 25534579

[20] Smith GE Housen P Yaffe K Ruff R Kennison RF Mahncke HW . A cognitive training program based on principles of brain plasticity: results from the improvement in memory with plasticity-based adaptive cognitive training (impact) study. J Am Geriatr Soc. (2009) 57:594–603. doi: 10.1111/j.1532-5415.2008.02167.x, PMID: 19220558 PMC4169294

[21] Mahncke HW Connor BB Appelman J Ahsanuddin ON Hardy JL Wood RA . Memory enhancement in healthy older adults using a brain plasticity-based training program: a randomized, controlled study. Proc Natl Acad Sci USA. (2006) 103:12523–8. doi: 10.1073/pnas.0605194103, PMID: 16888038 PMC1526649

[22] Butz M Gerriets T Sammer G El-Shazly J Tschernatsch M Huttner HB . Effects of postoperative cognitive training on neurocognitive decline after heart surgery: a randomized clinical trial. Eur J Cardiothorac Surg. (2022) 62:ezac251. doi: 10.1093/ejcts/ezac251 35415742

[23] Saleh AJ Tang G Hadi SM Yan L Chen M Duan K . Preoperative cognitive intervention reduces cognitive dysfunction in elderly patients after gastrointestinal surgery: a randomized controlled trial. Med Sci Monit. (2015) 21:798–805. doi: 10.12659/MSM.893359, PMID: 25782136 PMC4373157

[24] Chao-liang LOFZ. Effect of early cognitive behavior intervention on cognitive function in elderly patients after coronary artery bypass grafting. Chinese Journal of the Frontiers of Medical Science (Electronic Version). (2018) 10:96–9. doi: 10.12037/YXQY.2018.07-18

[25] O'Gara BP Mueller A Gasangwa DVI Patxot M Shaefi S Khabbaz K . Prevention of early postoperative decline: a randomized, controlled feasibility trial of perioperative cognitive training. Anesth Analg. (2020) 130:586–95. doi: 10.1213/ANE.0000000000004469, PMID: 31569161 PMC7154961

[26] Song Y Cui X Zhang Y Gao H Cai Q Mu Z. Home-based computerized cognitive training for postoperative cognitive dysfunction after lung transplantation in elderly population: a randomized controlled trial. J Nerv Ment Dis. (2019) 207:693–9. doi: 10.1097/NMD.0000000000001032 31356409

[27] Vlisides PE Das AR Thompson AM Kunkler B Zierau M Cantley MJ . Home-based cognitive prehabilitation in older surgical patients: a feasibility study. J Neurosurg Anesthesiol. (2019) 31:212–7. doi: 10.1097/ANA.0000000000000569, PMID: 30557230 PMC6652226

[28] Greaves D Astley J Psaltis PJ Lampit A Davis DH Ghezzi ES . The effects of computerised cognitive training on post-cabg delirium and cognitive change: a prospective randomised controlled trial. Delirium (Bielefeld, Germany). (2023) 1:67976. doi: 10.56392/001c.67976 36936538 PMC7614332

[29] Humeidan ML Reyes JC Mavarez-Martinez A Roeth C Nguyen CM Sheridan E . Effect of cognitive prehabilitation on the incidence of postoperative delirium among older adults undergoing major noncardiac surgery: the neurobics randomized clinical trial. JAMA Surg. (2021) 156:148–56. doi: 10.1001/jamasurg.2020.4371, PMID: 33175114 PMC7658803

[30] Wang L Liang W Wang B Guo M Zhou J Chen L . Transcutaneous electrical acupoint stimulation for reducing cognitive dysfunction in lumbar spine surgery: a randomized, controlled trail. Front Aging Neurosci. (2022) 14:1034998. doi: 10.3389/fnagi.2022.1034998, PMID: 36545028 PMC9760873

[31] Chen J Tu Q Miao S Zhou Z Hu S. Transcutaneous electrical acupoint stimulation for preventing postoperative nausea and vomiting after general anesthesia: a meta-analysis of randomized controlled trials. Int J Surg (London, England). (2020) 73:57–64. doi: 10.1016/j.ijsu.2019.10.036, PMID: 31704425

[32] Yuan S Zhang X Bo Y Li W Zhang H Jiang Q. The effects of electroacupuncture treatment on the postoperative cognitive function in aged rats with acute myocardial ischemia-reperfusion. Brain Res. (2014) 1593:19–29. doi: 10.1016/j.brainres.2014.10.005, PMID: 25446007

[33] Wei H Huang J Zhao F Xie Z Xia Z Gan J. Transcutaneous electrical acupoint stimulation improves postoperative cognitive function in senior patients undergoing video-assisted thoracoscopic surgery: a randomized controlled trial. Chin J Integr Med. (2022) 28:730–5. doi: 10.1007/s11655-022-3516-1, PMID: 35546221

[34] Wu Y Luo H. Effect of electrical acupoint stimulation on postoperative cognitive dysfunction after total intravenous anesthesia. Shanghai J Acupunct Moxib. (2020) 39:1161–5. doi: 10.13460/j.issn.1005-0957.2020.09.1161

[35] Li S Jiang H Liu W Yin Y Yin C Chen H . Transcutaneous electrical acupoint stimulation for the prevention of perioperative neurocognitive disorders in geriatric patients: a systematic review and meta-analysis of randomized controlled trials. Medicine (Baltimore). (2022) 101:e32329. doi: 10.1097/MD.0000000000032329, PMID: 36550918 PMC9771360

[36] Zhang T Ou L Chen Z Li J Shang Y Hu G. Transcutaneous electrical acupoint stimulation for the prevention of postoperative cognitive dysfunction: a systematic review and meta-analysis. Front Med (Lausanne). (2021) 8:756366. doi: 10.3389/fmed.2021.756366, PMID: 34938745 PMC8685241

[37] Zhang Penghui WX. Progress in the prevention of postoperative cognitive dysfunction in elderly patients by transcutaneous electrical stimulation of acupoints. Journal of Clinical Anesthesiology (China), (2018):720–2. doi: 10.12089/jca.2018.07.024,

[38] Chan MMY Yau SSY Han YMY. The neurobiology of prefrontal transcranial direct current stimulation (tdcs) in promoting brain plasticity: a systematic review and meta-analyses of human and rodent studies. Neurosci Biobehav Rev. (2021) 125:392–416. doi: 10.1016/j.neubiorev.2021.02.035, PMID: 33662444

[39] Lefaucheur J Antal A Ayache SS Benninger DH Brunelin J Cogiamanian F . Evidence-based guidelines on the therapeutic use of transcranial direct current stimulation (tdcs). Clin Neurophysiol. (2017) 128:56–92. doi: 10.1016/j.clinph.2016.10.087, PMID: 27866120

[40] Majdi A van Boekholdt L Sadigh-Eteghad S Mc LM. A systematic review and meta-analysis of transcranial direct-current stimulation effects on cognitive function in patients with Alzheimer's disease. Mol Psychiatry. (2022) 27:2000–9. doi: 10.1038/s41380-022-01444-7, PMID: 35115703

[41] Siegert A Diedrich L Antal A. New methods, old brains-a systematic review on the effects of tdcs on the cognition of elderly people. Front Hum Neurosci. (2021) 15:730134. doi: 10.3389/fnhum.2021.730134, PMID: 34776903 PMC8578968

[42] Xie Y Li Y Nie L Zhang W Ke Z Ku Y. Cognitive enhancement of repetitive transcranial magnetic stimulation in patients with mild cognitive impairment and early Alzheimer's disease: a systematic review and meta-analysis. Front Cell Dev Biol. (2021) 9:734046. doi: 10.3389/fcell.2021.734046, PMID: 34568342 PMC8461243

[43] Tao M Zhang S Han Y Li C Wei Q Chen D . Efficacy of transcranial direct current stimulation on postoperative delirium in elderly patients undergoing lower limb major arthroplasty: a randomized controlled trial. Brain Stimul. (2023) 16:88–96. doi: 10.1016/j.brs.2023.01.839, PMID: 36682718

[44] Wu T Li M Tian L Cong P Huang X Wu H . A modified mouse model of perioperative neurocognitive disorders exacerbated by sleep fragmentation. Exp Anim. (2023) 72:55–67. doi: 10.1538/expanim.22-0053, PMID: 36130912 PMC9978123

[45] Zhang F Qin Y Xie L Zheng C Huang X Zhang M. High-frequency repetitive transcranial magnetic stimulation combined with cognitive training improves cognitive function and cortical metabolic ratios in Alzheimer's disease. J Neural Transm (Vienna). (2019) 126:1081–94. doi: 10.1007/s00702-019-02022-y 31292734

[46] Zhao J Li Z Cong Y Zhang J Tan M Zhang H . Repetitive transcranial magnetic stimulation improves cognitive function of Alzheimer's disease patients. Oncotarget. (2017) 8:33864–71. doi: 10.18632/oncotarget.13060, PMID: 27823981 PMC5464918

[47] Rabey JM Dobronevsky E Aichenbaum S Gonen O Marton RG Khaigrekht M. Repetitive transcranial magnetic stimulation combined with cognitive training is a safe and effective modality for the treatment of Alzheimer's disease: a randomized, double-blind study. J Neural Transm (Vienna). (2013) 120:813–9. doi: 10.1007/s00702-012-0902-z 23076723

[48] Lee SS Lo Y Verghese J. Physical activity and risk of postoperative delirium. J Am Geriatr Soc. (2019) 67:2260–6. doi: 10.1111/jgs.16083, PMID: 31368511 PMC6861610

[49] Trubnikova OA Tarasova IV Moskin EG Kupriyanova DS Argunova YA Pomeshkina SA . Beneficial effects of a short course of physical prehabilitation on neurophysiological functioning and neurovascular biomarkers in patients undergoing coronary artery bypass grafting. Front Aging Neurosci. (2021) 13:699259. doi: 10.3389/fnagi.2021.699259, PMID: 34955803 PMC8704127

[50] Yanagisawa T Tatematsu N Horiuchi M Migitaka S Yasuda S Itatsu K . Preoperative low physical activity is a predictor of postoperative delirium in patients with gastrointestinal cancer: a retrospective study. Asian Pac J Cancer Prev. (2022) 23:1753–9. doi: 10.31557/APJCP.2022.23.5.1753, PMID: 35633561 PMC9587851

[51] Chapman SB Aslan S Spence JS Defina LF Keebler MW Didehbani N . Shorter term aerobic exercise improves brain, cognition, and cardiovascular fitness in aging. Front Aging Neurosci. (2013) 5:75. doi: 10.3389/fnagi.2013.00075, PMID: 24282403 PMC3825180

[52] Cassilhas RC Tufik S de Mello MT. Physical exercise, neuroplasticity, spatial learning and memory. Cell Mol Life Sci. (2016) 73:975–83. doi: 10.1007/s00018-015-2102-0 26646070 PMC11108521

[53] Cho M Song S Ryu C. Sleep disturbance strongly related to the development of postoperative delirium in proximal femoral fracture patients aged 60 or older. Hip & Pelvis. (2020) 32:93–8. doi: 10.5371/hp.2020.32.2.93, PMID: 32566540 PMC7295614

[54] Hu R Jiang X Chen J Zeng Z Chen XY Li Y . Non-pharmacological interventions for sleep promotion in the intensive care unit. Cochrane Database Syst Rev. (2015) 2015:CD008808. doi: 10.1002/14651858.CD008808.pub2, PMID: 26439374 PMC6517220

[55] Litton E Carnegie V Elliott R Webb SAR. The efficacy of earplugs as a sleep hygiene strategy for reducing delirium in the icu: a systematic review and meta-analysis. Crit Care Med. (2016) 44:992–9. doi: 10.1097/CCM.0000000000001557, PMID: 26741578

[56] Kamdar BB King LM Collop NA Sakamuri S Colantuoni E Neufeld KJ . The effect of a quality improvement intervention on perceived sleep quality and cognition in a medical icu. Crit Care Med. (2013) 41:800–9. doi: 10.1097/CCM.0b013e3182746442, PMID: 23314584 PMC3579000

[57] Menger J Urbanek B Skhirtladze-Dworschak K Wolf V Fischer A Rinösl H . Earplugs during the first night after cardiothoracic surgery may improve a fast-track protocol. Minerva Anestesiol. (2018) 84:49–57. doi: 10.23736/S0375-9393.17.11758-X, PMID: 28726359

[58] Leong RW Davies LJ Fook-Chong S Ng SY Lee YL. Effect of the use of earplugs and eye masks on the quality of sleep after major abdominal surgery: a randomised controlled trial. Anaesthesia. (2021) 76:1482–91. doi: 10.1111/anae.15468, PMID: 33881774

[59] Walsh S Causer R Brayne C. Does playing a musical instrument reduce the incidence of cognitive impairment and dementia? A systematic review and meta-analysis. Aging Ment Health. (2021) 25:593–601. doi: 10.1080/13607863.2019.1699019, PMID: 31814445

[60] Khan SH Xu C Purpura R Durrani S Lindroth H Wang S . Decreasing delirium through music: a randomized pilot trial. Am J Crit Care. (2020) 29:e31–8. doi: 10.4037/ajcc2020175, PMID: 32114612 PMC7666845

[61] Xin WNQY. Effect of 49elaxation training combined with baker's cognitive therapy on cardiac muscle contraction reserve and cognitive status in patients with pocd after aortic dissection. J Int Psychiatry. (2022) 49:175–8. doi: 10.13479/j.cnki.jip.2022.01.024

[62] Golubovic J Neerland BE Aune D Baker FA. Music interventions and delirium in adults: a systematic literature review and meta-analysis. Brain Sci. (2022) 12:12. doi: 10.3390/brainsci12050568, PMID: 35624955 PMC9138821

[63] Goodman CS Shatz CJ. Developmental mechanisms that generate precise patterns of neuronal connectivity. Cell. (1993) 72:77–98. doi: 10.1016/S0092-8674(05)80030-3 8428376

[64] Baroncelli L Braschi C Spolidoro M Begenisic T Sale A Maffei L. Nurturing brain plasticity: impact of environmental enrichment. Cell Death Differ. (2010) 17:1092–103. doi: 10.1038/cdd.2009.193, PMID: 20019745

[65] Hua M Min J. Postoperative cognitive dysfunction and the protective effects of enriched environment: a systematic review. Neurodegener Dis. (2020) 20:113–22. doi: 10.1159/000513196, PMID: 33601385

[66] Duan S Liao Y Tang Y Zhang B Peng M Tong J . Short-term perioperative cognitive therapy combined with rehabilitation exercise reduces the incidence of neurocognitive disorder in elderly patients: a randomized controlled trial. Minerva Anestesiol. (2022) 88:145–55. doi: 10.23736/S0375-9393.21.15877-8, PMID: 35315627

[67] Linying P. Short-term exercise reduced the incidence of postoperative cognitive dysfuction (pocd) and inflammatory cytokines in the elderly patients. Central South University (China) (2014).

[68] Huson K Stolee P Pearce N Bradfield C Heckman GA. Examining the hospital elder life program in a rehabilitation setting: a pilot feasibility study. BMC Geriatr. (2016) 16:140. doi: 10.1186/s12877-016-0313-3, PMID: 27431505 PMC4949744

[69] Yue J Tabloski P Dowal SL Puelle MR Nandan R Inouye SK. Nice to help: operationalizing national institute for health and clinical excellence guidelines to improve clinical practice. J Am Geriatr Soc. (2014) 62:754–61. doi: 10.1111/jgs.12768, PMID: 24697606 PMC4020349

[70] Inouye SK Bogardus STJ Charpentier PA Leo-Summers L Acampora D Holford TR . A multicomponent intervention to prevent delirium in hospitalized older patients. N Engl J Med. (1999) 340:669–76. doi: 10.1056/NEJM199903043400901 10053175

[71] Hshieh TT Yang T Gartaganis SL Yue J Inouye SK. Hospital elder life program: systematic review and meta-analysis of effectiveness. Am J Geriatr Psychiatry. (2018) 26:1015–33. doi: 10.1016/j.jagp.2018.06.007, PMID: 30076080 PMC6362826

[72] Wang Y Yue J Xie D Carter P Li Q Gartaganis SL . Effect of the tailored, family-involved hospital elder life program on postoperative delirium and function in older adults: a randomized clinical trial. JAMA Intern Med. (2020) 180:17–25. doi: 10.1001/jamainternmed.2019.4446, PMID: 31633738 PMC6806427

[73] Liang C Chu C Hsu Y Chou M Wang Y Lin Y . Effects of modified version of the hospital elder life program on post-discharge cognitive function and activities of daily living among older adults undergoing total knee arthroplasty. Arch Gerontol Geriatr. (2021) 93:104284. doi: 10.1016/j.archger.2020.104284, PMID: 33157357

[74] Kehlet H. Enhanced postoperative recovery: good from afar, but far from good? Anaesthesia. (2020) 75:e54–61. doi: 10.1111/anae.14860, PMID: 31903577

