# Supplementary figures and images for: The research progress of perioperative non-pharmacological interventions on postoperative cognitive dysfunction: a narrative review
Source: Front Neurol. 2024 May 1;15:1369821. doi: 10.3389/fneur.2024.1369821 (PMC11094646; doi:10.3389/fneur.2024.1369821)

Additional material 1: The results of the Pubmed search.


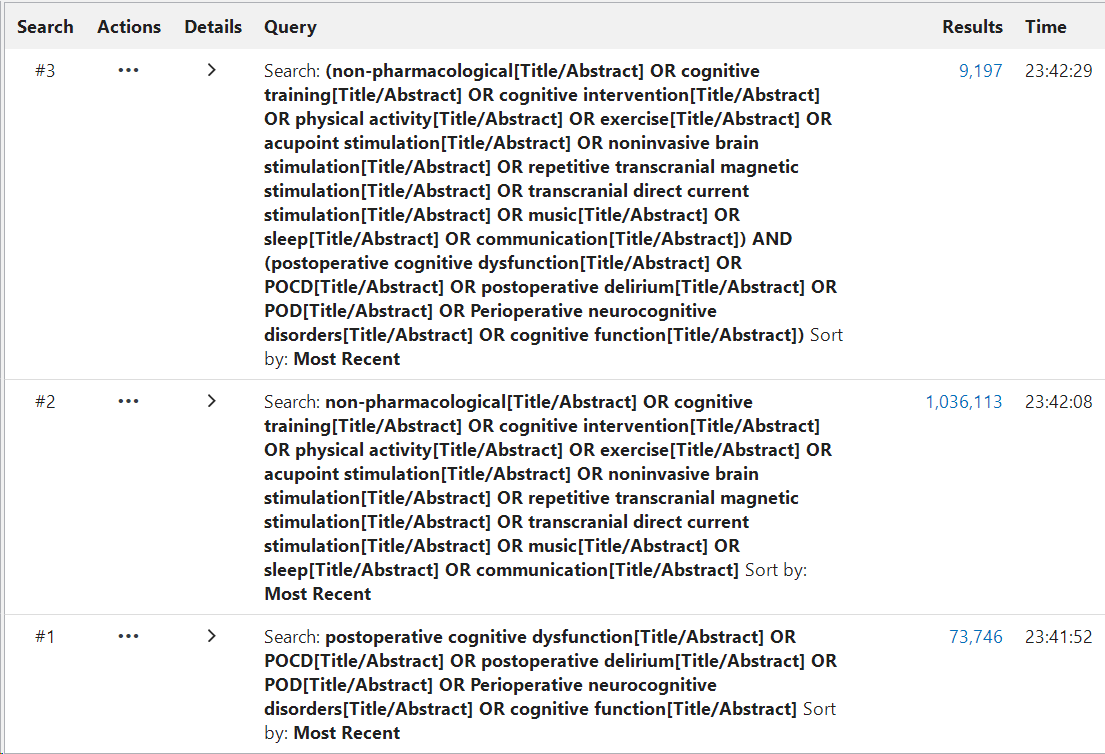

Supplement: Supplementary file 1 [file Table_1.DOCX]
